# Comparative studies of versatile extracellular proteolytic activities of lactic acid bacteria and their potential for extracellular amino acid productions as feed supplements

**DOI:** 10.1186/s40104-019-0323-z

**Published:** 2019-03-07

**Authors:** Ye Heng Lim, Hooi Ling Foo, Teck Chwen Loh, Rosfarizan Mohamad, Norhani Abdullah

**Affiliations:** 1 Institute of Bioscience, Universiti Putra Malaysia, 43400 UPM, Serdang, Selangor Malaysia; 2 Department of Bioprocess Technology, Faculty of Biotechnology and Biomolecular Sciences, Universiti Putra Malaysia, 43400 UPM, Serdang, Selangor Malaysia; 3 Department of Animal Sciences, Faculty of Agriculture, Universiti Putra Malaysia, 43400 UPM, Serdang, Selangor Malaysia; 4 Institute of Tropical Agriculture and Food Security, Universiti Putra Malaysia, 43400 UPM, Serdang, Selangor Malaysia; 5 Institute of Tropical Forestry and Forest Products, Universiti Putra Malaysia, 43400 UPM, Serdang, Selangor Malaysia

**Keywords:** Amino acid, Bio-agent, Extracellular proteolytic activity, Feed supplement, Lactic acid bacteria, *Lactobacillus*, *Pediococcus*

## Abstract

**Background:**

Increasing understanding on the functions of amino acids (AA) has led to new commercial applications and expansion of the worldwide markets. However, the current technologies rely heavily on non-food grade microorganism and chemical synthesis for the production of AA. Several studies reported that lactic acid bacteria (LAB) have the capability of producing AA owing to their well-established proteolytic system and amino acid biosynthesis genes. Hence, the objectives of this study were to explore the extracellular proteolytic activity of LAB isolated from various Malaysian fermented foods and their potential to produce AA extracellularly as feed supplements.

**Results:**

All the studied LAB isolates were versatile extracellular protease producers, whereby extracellular protease activities were detected from acidic to alkaline pH (pH 5, pH 6.5, pH 8) using qualitative and quantitative proteolytic assays. The highest proteolytic activity at pH 5 (15.76 U/mg) and pH 8 (19.42 U/mg) was achieved by *Lactobacillus plantarum* RG14, while *Lactobacillus plantarum* RS5 exhibited the highest proteolytic activity of 17.22 U/mg at pH 6.5. As for the results of AA production conducted in de Man, Rogosa and Sharpe medium and analysed by high pressure liquid chromatography system, all LAB isolates were capable of producing an array of AA. Generally, *Pediococcus* sp. showed greater ability for AA production as compared to *Lactobacillus* sp. Moreover, the studied LAB were able to produce a few major feed supplement AA such as methionine, lysine, threonine and tryptophan. *P. pentosaceus* TL-3 recorded the highest methionine and threonine productivity of 3.72 mg/L/h and 5.58 mg/L/h respectively. However, *L. plantarum* I-UL4 demonstrated a lysine productivity of 1.24 mg/L/h, while *P. acidilactici* TP-6 achieved up to 1.73 mg/L/h of tryptophan productivity.

**Conclusion:**

All the 17 studied LAB isolates possessed versatile extracellular proteolytic system and have vast capability of producing various amino acids including a few major feed supplement AA such as methionine, lysine, threonine and tryptophan. Despite AA production was strain dependent, the studied LAB isolates possessed vast potential and can be exploited further as a bio-agent or an alternative amino acids and bioactive peptide producers.

## Background

Amino acid (AA) are building blocks of proteins, which are one of the most essential components of life [1]. Increasing understanding on the functions and properties of amino acid had led to increasing commercial interest and diverse commercial applications. Over the past two decades, the development of AA industry was vibrant and revolved around AA for feed supplements, constituting 56% of the total AA market. Meanwhile, the remaining 44% were mainly used in food, pharmaceutical, agriculture and cosmetics sectors [2].

AA supplementation is commonly practised in livestock industry due to the limiting quantities of essential AA in the animal feeds, which may lead to growth impairment and poor productivity of livestock. Hence, AA supplementation is crucial to fulfil the limiting AA requirement of the animals and ensure proper function of the animals’ biological system [3]. Besides, supplementation of AA allows the use of feed formula with low level of crude protein, which is economically advantageous and contributes greatly to relief crude protein deficiency [4]. The most commonly used AA in feed supplements include *L*-lysine, *DL*-methionine, *L*-threonine and *L*-tryptophan, due to their pronounced effects on livestock growth performance and meat quality [5–10]. Apart from improving the well-being and growth performance of livestock, supplementation of AA in the animals’ diet could effectively improve nitrogen utilisation and minimise nitrogen excretion [11].

Generally, AA can be produced through three different methods, namely extraction, chemical synthesis and microbial methods. At present, microbial methods are conveniently used for the production of most AA due to its economic and ecological advantages [12]. The most commonly employed AA producer in the industry are genetically modified strains of *Corynebacterium glutamicum* and *Escherichia coli* [2]. However, industries may come to reluctant when using these microorganisms due to the pathogenicity and the non-food grade status [13]. Moreover, utilisation of genetically engineered bacteria for the production of AA had its share of controversies as the use of genetically modified *C. glutamicum* for the production of AA has been linked to over thousand cases of a deadly syndrome, known as eosinophila myalgia syndrome (EMS). This has urged for the search of food grade microorganisms as an alternative AA producer.

Lactic acid bacteria (LAB) appear to be an excellent alternative candidate for AA production, due to their non-pathogenic nature and reputation as “Generally Recognised as Safe (GRAS)” bacteria [14]. LAB are one of the most commonly employed probiotics [15–17], attributing to their important role in improving the gastrointestinal health [18, 19] of the host by producing antimicrobial compounds [19–21] and reducing harmful microorganisms in the intestine [22]. Several reports suggested that LAB possess a well-established proteolytic system, which may contribute to the hydrolysis of complex protein to release free AA [23–26]. Additionally, the presence of active AA biosynthesis pathway and the relevant genes responsible for AA production have been reported for LAB [27, 28].

Despite extensive reports on proteolytic activity of LAB, the documentation on application of LAB for the production of AA are very limited. Hence, the objectives of this study were to explore the extracellular proteolytic activity of LAB isolated from various Malaysian fermented foods and to evaluate their ability to produce AA particularly extracellularly for feed supplements.

## Methods

### Microorganism and maintenance

Seventeen LAB isolates [9 *Lactobacillus plantarum*: TL-1, TL-2, TP-2, TP-5 (isolated from *tempeh*-fermented soybean cake), I-UL4 (isolated from *tapai ubi*-fermented cassava), RI 11, RG 11, RG 14, RS 5 (isolated from *ikan rebus*-steam fish); 6 *Pediococcus pentosaceus*: B12m9 (isolated from *budu*-fermented fish sauce), TB-1, TL-3, TP-3, TP-4, TP-8 (isolated from *tempeh*-fermented soybean cake); 2 *Pediococcus acidilactici*: TB-2, TP-6 (isolated from *tempeh*-fermented soybean cake)] that previously isolated from Malaysian fermented foods were obtained from the Laboratory of Industrial Biotechnology, Department of Bioprocess Technology, Faculty of Biotechnology and Biomolecular Sciences, Universiti Putra Malaysia [29–31]. The LAB cultures were maintained and revived as described by Foo et al. [32].

### Determination of proteolytic activity

#### Preparation of extracellular enzyme

The preparation of extracellular enzymes were performed according to the method of Thung [31] with slight modification, where a smaller inoculum size of 1% (*v*/*v*) was used in this study. In brief, the active LAB culture was washed once with sterile 0.85% (*w*/*v*) NaCl (Merck, Germany) solution and adjusted to 10^9^ CFU/mL to be used as inoculum. A volume of 1% (*v*/*v*) of the adjusted LAB culture was inoculated into 10 mL de Man, Rogosa and Sharpe (MRS) medium (Merck, Germany) and incubated at 30 °C for 10 h, followed by centrifugation at 10,000×*g* for 15 min to separate the biomass from supernatant. The supernatant was then collected and filtered through a 0.2-μm cellulose acetate membrane (Sartorius Stedim, Germany) to obtain cell-free-supernatant (CFS), which was used as extracellular enzyme for the determination of extracellular proteolytic activity.

#### Qualitative determination of proteolytic activity

The proteolytic activity of the LAB isolate was detected qualitatively by using skim milk agar hydrolysis method [31] with minor modification, where log phase (10 h) culture was used in the assay. A loopful of 10 h LAB culture with a cell population of 10^9^ CFU/mL was streaked on skim milk agar containing 1% (*w*/*v*) skim milk (Merck, Germany) and incubated at 30 °C for 48 h. Proteolytic activity was indicated by the occurrence of clear hydrolysis zone. All analyses were performed in triplicates.

#### Effect of pH on extracellular proteolytic activity

Skim milk agar well diffusion (SMAWD) assay was used to determine the active pH range of the extracellular proteases produced by the LAB isolates. Three different pH conditions provided by 0.1 mol/L sodium acetate buffer (pH 5.0), 0.1 mol/L sodium phosphate buffer (pH 6.5) and 0.1 mol/L Tris-HCl buffer (pH 8.0) were used in the assay [31]. The CFS was mixed with the respective buffer at 1:1 ratio. A volume of 20 μL of the buffered CFS was then inoculated into the pre-punched well on skim milk agar and incubated for 48 h at 30 °C, followed by observation for clear hydrolysis zone. Buffer of respective pH without CFS was used as control. All analyses were performed in triplicates.

#### Quantification of extracellular proteolytic activity

The extracellular protease activity was quantified under 3 different pH conditions as described by Thung [31] with minor modification. In brief, 0.25 mL of CFS was added to 0.5 mL of buffer containing 0.5% (*w*/*v*) sulphanilamide azocasein (Sigma Aldrich, USA) and incubated at 37 °C for 30 min. Next, 0.75 mL of 10% (*w*/*v*) trichloroacetic acid (Merck, Germany) was added and incubated at room temperature for 30 min to terminate the reaction. The precipitate was removed by centrifugation at 12,000×*g* for 10 min. A volume of 0.6 mL of the supernatant was mixed with 0.6 mL of 1 mol/L NaOH (Merck, Germany) and incubated for 15 min at room temperature prior to measuring its absorbance at 450 nm. The control of the assay was prepared by substituting the CFS and substrate with buffer respectively. One unit (U/mg) of specific protease activity was defined as the amount of enzyme capable of hydrolysing sulphanilamide-azocasein to produce 0.001 change in absorbance per minute of incubation time per mg of protein under the assay condition. All analyses were performed in triplicates.

#### Protein content determination

Protein content of the CFS was determined by using Bradford method [33], whereby bovine serum albumin (Sigma Aldrich, USA) was used as reference. In brief, 0.5 mL of appropriately diluted CFS was mixed with 0.5 mL of Bradford reagent (Sigma Aldrich, USA) and incubated for 5 min at 4 °C. The absorbance was measured at 595 nm using a Varian Cary 50 spectrophotometer (Agilent Technologies, USA) and the protein content was quantified based on the standard curve of bovine serum albumin. All analyses were performed in triplicates.

#### Production of amino acids by LAB isolates

The production of AA was conducted as described by Norfarina et al. [34] with modifications. Briefly, the active LAB culture was washed once with 0.85% (*w*/*v*) NaCl solution and adjusted to a cell population of 10^9^ CFU/mL. A volume of 10% (*v*/*v*) of the adjusted culture was then inoculated into MRS medium and incubated at 30 °C for 24 h. Samples were collected at every 2 h intervals and the CFS was used for the determination of AA production profile. The MRS medium without inoculum was served as control.

#### Determination of amino acid production profile

The AA production profile of CFS was analysed as described by Henderson et al. [35] by using Agilent 1100 high performance liquid chromatograph (HPLC) (Agilent Technologies, USA). Derivatisation of AA was performed by using o-phthalaldehyde (OPA) and 9- fluorenylmethyl chloroformate (FMOC). The derivatised AA were separated on a Zorbax Eclipse Plus C18 reverse phase column (4.6 mm × 150 mm, 3.5 μm) (Agilent Technologies, USA). The bound AA were eluted with 40 mmol/L sodium dihydrogen phosphate monohydrate (NaH_2_PO_4_·H_2_O) adjusted to pH 7.8 and a methanol-acetonitrile-deionised water mixture (9:9:2) at a flow rate of 2 mL/min. The OPA, FMOC and NaH_2_PO_4_·H_2_O were analytical grade while the methanol and acetonitrile were HPLC grade that purchased from Merck. The eluted derivatised AA were detected by a fluorescence detector (Agilent Technologies, USA) at the excitation/emission wavelengths of 340/450 nm for primary AA and 266/305 nm for secondary AA. The AA concentration was quantified by referring to the calibration curve constructed by using AA standard (Sigma Aldrich, USA). The production of AA was calculated by deducting the highest concentration of each AA with their respective initial concentration. All analyses were performed in triplicates.

#### Statistical analysis

The results were analysed by one-way analysis of variance (ANOVA) using Statistical Analysis System (SAS 9.1, USA). Duncan’s Multiple Range Test System was used to compare the significant difference between the mean at *P* < 0.05.

## Results & discussion

### Qualitative determination of extracellular proteolytic activity

**Fig. 1:**
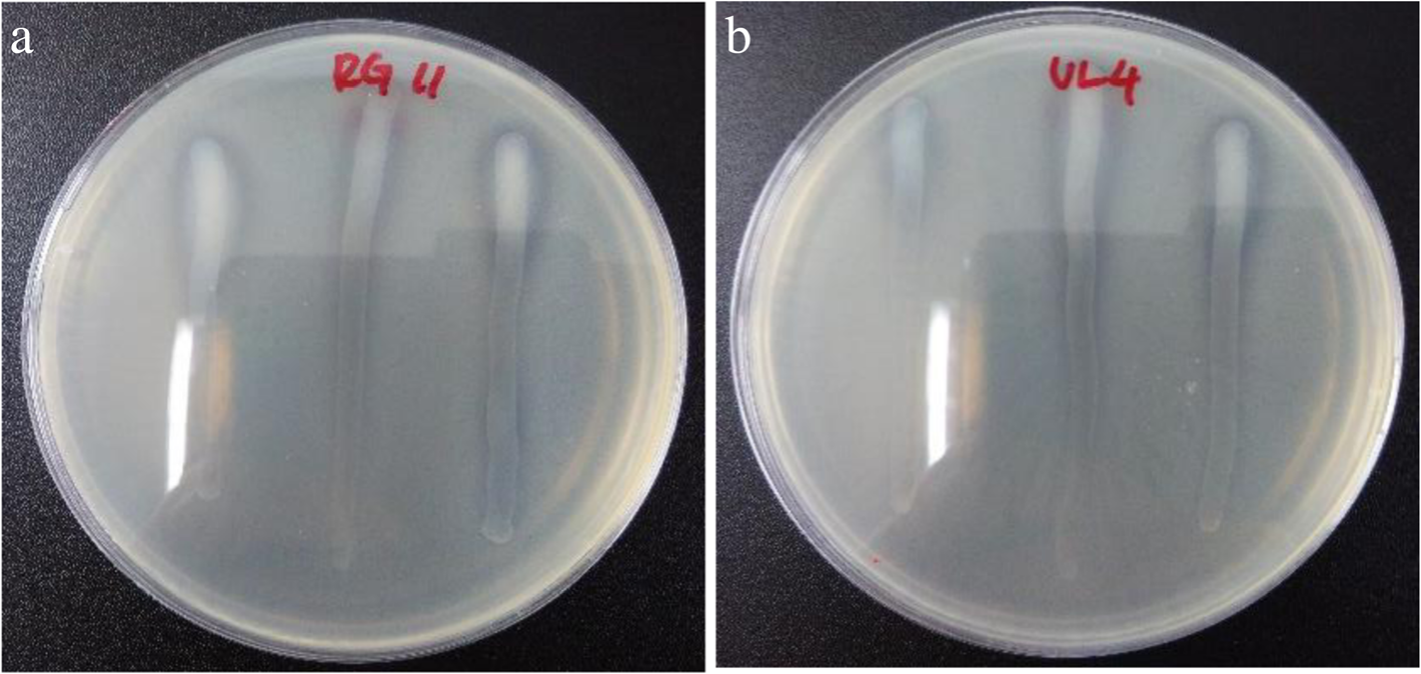
Representative of hydrolysis zone formation obtained in skim milk agar hydrolysis assay. **a** *L. plantarum* RG 11, (**b**) *L. plantarum* I-UL4

The proteolytic activity of LAB had been studied extensively due to their industrial importance [36] and essential role in ensuring the survival of the bacteria [37, 38]. In the present study, the ability of the 17 LAB isolated from Malaysian fermented foods to produce and secrete extracellular proteolytic enzymes was determined qualitatively by using skim milk agar hydrolysis assay. Results obtained in this study revealed that all the 17 studied LAB isolates were capable to produce clear hydrolysis zone on skim milk agar as illustrated in Fig. 1. This inferred that all the 17 tested LAB isolates were capable to produce and secrete extracellular proteolytic enzymes, which is responsible to hydrolyse whitish opaque colour casein molecules into colourless peptide fragments, thereby producing clear zone around the culture. Pailin et al. [39] reported similar finding, where all studied LAB isolates demonstrated ability to form clear zone on skim milk. Likewise, majority of the LAB isolated from Algerian goat’s milk [40] and Egyptian Ras cheese [41] have demonstrated the ability to produce clear hydrolysis zone on skim milk agar. This implied that majority of LAB possessed extracellular proteolytic activity.

### Effects of pH on extracellular proteolytic activity

The effects of pH on extracellular proteolytic activity of the LAB isolates were investigated by using SMAWD assay in three different pH conditions that resembled acidic (pH 5), near neutral (pH 6.5) and alkaline (pH 8) conditions. Generally, proteolytic enzymes can be categorised into three distinct groups, namely acidic, neutral and alkaline proteases based on their active pH range [42]. Results obtained in the current study demonstrated that the CFS of the 17 LAB isolates have the capability to produce clear zone of hydrolysis under three different pH conditions, where occurrence of halo was observed around the well containing CFS adjusted to different pH conditions. The diameter of clear hydrolysis zone produced by CFS of the 17 LAB isolates under 3 different pH conditions are summarised in Table 1. Development of clear hydrolysis zone by CFS of the LAB suggested that the proteolytic enzymes produced by the isolates were secreted and active extracellularly. This is in agreement with the findings reported by Beganovic et al. [43], where formation of clear hydrolysis zone around the skim milk agar well inoculated with actively growing LAB cell was observed, indicating that LAB were capable to produce and secrete extracellular proteolytic enzymes. The presence of extracellular proteolytic activity in *Lactobacillus acidophilus*, *Bifidobacterium* sp., *L. casei, Streptococcus thermophilus*, and *Pediococcus acidilactici* was also well documented [44, 45]. However, the occurrence of hydrolysis zone under different pH conditions (Table 1) indicated that the extracellular proteases produced by the studied LAB isolates were active from acidic to alkaline pH, and hence implied that they were versatile producer for extracellular proteolytic enzymes. Similar findings were reported by Addi and Guessas [46], where proteolytic activity was detected in CFS of *Lactococcus* sp. over a broad pH conditions ranging from pH 5.5 to pH 8 with the highest proteolytic activity detected at near neutral condition (pH 7.2).

**Table 1:** Diameter of hydrolysis zone formed by CFS of LAB isolates in SMAWD assay

Isolates	pH 5*	Level**	pH 6.5	Level	pH 8	Level
*P. pentosaceus* B12m9	1.17 ± 0.03^Aa^	+++	1.00 ± 0.00^ABCb^	++	0.90 ± 0.00^CDc^	+
*P. pentosaceus* TB-1	1.20 ± 0.00^Aa^	+++	0.87 ± 0.03^Eb^	+	0.90 ± 0.00^CDb^	+
*P. pentosaceus* TL-3	1.17 ± 0.03^Aa^	+++	0.97 ± 0.03^BCDb^	+	0.97 ± 0.03^BCb^	+
*P. pentosaceus* TP-3	1.13 ± 0.03^ABa^	+++	0.90 ± 0.00^DEb^	+	0.93 ± 0.03^BCb^	+
*P. pentosaceus* TP-4	1.13 ± 0.03^ABa^	+++	0.93 ± 0.03^CDEb^	+	0.90 ± 0.00^CDb^	+
*P. pentosaceus* TP-8	1.13 ± 0.03^ABa^	+++	0.93 ± 0.03^CDEb^	+	0.90 ± 0.00^CDb^	+
*P. acidilactici* TB-2	1.13 ± 0.03^ABa^	+++	0.93 ± 0.03^CDEb^	+	0.83 ± 0.03^DEb^	+
*P. acidilactici* TP-6	1.10 ± 0.00^ABa^	+++	0.90 ± 0.00^DEb^	+	0.80 ± 0.00^Ec^	+
*L. plantarum* TL-1	1.13 ± 0.03^ABa^	+++	0.93 ± 0.03^CDEb^	+	0.90 ± 0.00^CDb^	+
*L. plantarum* TL-2	1.20 ± 0.00^Aa^	+++	1.00 ± 0.00^ABCb^	++	1.00 ± 0.00^Bb^	++
*L. plantarum* TP-2	1.13 ± 0.03^ABa^	+++	1.03 ± 0.03^ABa^	++	1.07 ± 0.03^Aa^	++
*L. plantarum* TP-5	1.20 ± 0.00^Aa^	+++	1.07 ± 0.03^Ab^	++	1.10 ± 0.00^Ab^	+++
*L. plantarum* RI11	1.13 ± 0.03^ABa^	+++	1.00 ± 0.00^ABCb^	++	0.90 ± 0.00^CDv^	+
*L. plantarum* RG11	1.07 ± 0.03^Ba^	++	0.93 ± 0.03^CDEb^	+	0.93 ± 0.03^BCb^	+
*L. plantarum* RG14	1.17 ± 0.03^Aa^	+++	0.97 ± 0.03^BCDb^	+	0.93 ± 0.03^BCb^	+
*L. plantarum* RS5	1.07 ± 0.03^Ba^	++	0.93 ± 0.03^CDEb^	+	0.93 ± 0.03^BCb^	+
*L. plantarum* I-UL4	1.13 ± 0.03^ABa^	+++	0.97 ± 0.03^BCDb^	+	0.93 ± 0.03^BCb^	+

*Values are mean ± standard error of the mean (SEM), *n* = 3. Mean ± SEM within the same column that share a similar capital letter superscript (A-E) are not significantly different (*P* > 0.05) while means within the same row that bear a common small letter superscript (a-c) indicate no significant difference (*P* > 0.05)

**The level of proteolytic activity was assigned based on the diameter of clear hydrolysis zone such that: ‘+’ indicates < 1.0 cm; ‘++’ indicates ≥1.0 cm but < 1.10 cm whereas ‘+++’ indicates ≥1.10 cm

The extracellular proteolytic activity of the LAB isolates were also determined semi-quantitatively by measuring the diameter of clear hydrolysis zone produced by the pH adjusted CFS of the LAB isolates. A larger clear hydrolysis zone indicated the occurrence of higher proteolytic activity. Results obtained in current study showed that all the 17 LAB isolates demonstrated significantly higher (*P* < 0.05) extracellular proteolytic activity under acidic condition, except *L. plantarum* TP-2, which revealed no significant difference (*P* > 0.05) between the extracellular proteolytic activities in 3 different pH conditions (Table 1). Similar findings were reported by Rollán et al. [47, 48], where the extracellular proteases of *Leuconostoc oenos* showed higher proteolytic activity at acidic pH condition. In addition, de Giori et al. [49] also demonstrated that the proteolysis of *Lactococci* and *Lactobacillus casei* occurred optimally between pH 4.8–5.6. The higher extracellular proteolytic activity at acidic pH condition could be attributed to the acidophilic nature of LAB [50]. Extracellular enzymes of acidophilic microorganisms are often optimally active at low pH [51]. Nevertheless, the extracellular proteolytic activity for most of the LAB isolates at pH 6.5 and pH 8 were not significantly different (*P* > 0.05), except for *P. pentosaceus* B12m9, *P. acidilactici* TP-6 and *L. plantarum* RI11, which demonstrated significantly higher (*P* < 0.05) extracellular proteolytic activity at pH 6.5.

Among the 17 tested LAB isolates, the highest extracellular proteolytic activity at pH 5 was detected in *P. pentosaceus* TB-1, *L. plantarum* TL-2 and *L. plantarum* TP-5, where the largest clear hydrolysis zone with a diameter of 1.2 cm was observed. However, they were not significantly different (*P* > 0.05) as compared to other LAB isolates, except for *L. plantarum* RG11 and *L. plantarum* RS5. In comparison, the highest extracellular proteolytic activity at pH 6.5 was achieved by *L. plantarum* TP-5. Nevertheless, it was not significantly different (*P* > 0.05) as compared to *P. pentosaceus* B12m9, *L. plantarum* TP-2 and *L. plantarum* RI11. On the other hand, the highest alkaline protease activity was recorded by *L. plantarum* TP-5, followed by *L. plantarum* TP-2. Generally, *L. plantarum* TP-5 exhibited the highest extracellular proteolytic activity, where the largest clear hydrolysis zone was observed under all the 3 pH conditions, followed by *L. plantarum* TL-2 and *L. plantarum* TP-2.

### Quantification of extracellular proteolytic activity

**Fig. 2:**
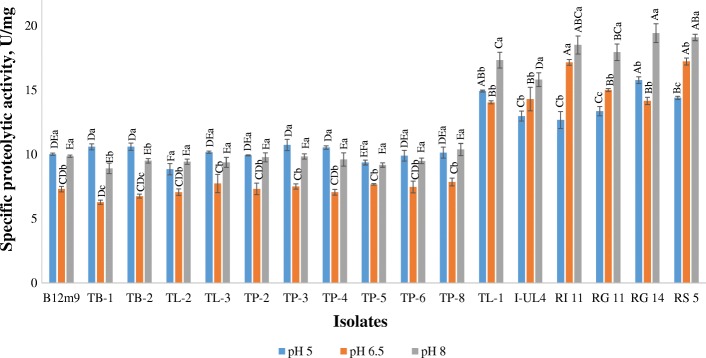
Extracellular proteolytic activity of LAB isolates at pH 5, pH 6.5 and pH 8. Values are mean ± standard error mean (SEM), *n* = 3. Vertical bars represent SEM. Values bearing different capital letter alphabets (A-F) among bacteria isolates are significant different (*P* < 0.05) while values sharing different small letter alphabet (a-c) among various pH are significant different (*P* < 0.05)

The extracellular proteolytic activity of the 17 LAB isolates was further quantified by using sulphanilamide-azocasein as substrate under three different pH conditions. Results that obtained in the quantitative assay (Fig. 2) were in agreement with the results obtained in SMAWD assay, whereby all the 17 tested LAB isolates exhibited extracellular proteolytic activity in three different pH conditions (pH 5, pH 6.5 and pH 8 respectively). In general, *Lactobacillus* sp. demonstrated comparatively higher extracellular proteolytic activity as compared to *Pediococcus* sp. The extracellular proteolytic activity of *Lactobacillus* sp. was between 7 U/mg to 19 U/mg, whereas the extracellular proteolytic activity of *Pediococcus* sp. were between 6 U/mg to 11 U/mg, indicating the highest extracellular proteolytic activity of *Pediococcus* sp. was approximately half of those recorded by *Lactobacillus* sp. A study conducted by Pailin et al. [39] also showed that *Lactobacillus* sp. exhibited comparatively higher extracellular proteolytic activity as compared to other tested LAB species. This could be attributed to the disruption of amino acid synthesis pathway in *Lactobacillus* sp., which is compensated with pronounced proteolytic activity [52].

Each LAB isolate produced different strength of extracellular proteolytic activity under different pH conditions, indicating that the proteolytic activity of LAB was strain dependent. For instances, 2 of the tested LAB isolates (*P. pentosaceus* TB-1 & *P. acidilactici* TB-2) demonstrated significantly higher (*P* < 0.05) extracellular proteolytic activity at pH 5, which is in agreement with de Giori et al. [49] who has reported that higher proteolytic activity was detected in LAB under acidic condition and a marked reduction in proteolytic activity of LAB was detected when the pH was near neutral at pH 6.6. Nonetheless, 6 out of the 17 studied LAB isolates (*L. plantarum* TL-1, I-UL4, RI 11, RG 11, RG 14 & RS 5) exhibited significantly higher (*P* < 0.05) extracellular proteolytic activity in the alkaline environment in this study. Production of alkaline proteases by various LAB including *L. plantarum* [53], *P. pentosaceus* [54], *L. helveticus* [55], and *S. thermophiles* [56] was well documented. Interestingly, the other 9 LAB isolates displayed comparably high level of extracellular proteolytic activity in both acidic and alkaline conditions as compared to their proteolytic activity at pH 6.5. Results obtained in the quantitative assay suggested that pH exerted a great impact on the activity of extracellular proteases produced by the studied LAB. The pronounced effect of pH on proteolytic activity was probably due to the alteration of hydrogen-ion equilibrium, which consequently modified the active structure of the enzyme or altered the protonation state of the substrate, hence affected the overall proteolytic activity [57].

Among the 17 studied LAB isolates, *L. plantarum* RG14 demonstrated the highest extracellular proteolytic activity in both acidic and alkaline environment with a specific extracellular proteolytic activity of 15.76 U/mg and 19.42 U/mg respectively. However, the extracellular proteolytic activity recorded by *L. plantarum* RG14 in acidic condition was not significantly different (*P* > 0.05) from *L. plantarum* TL-1. Meanwhile, *L. plantarum* RI11 and *L. plantarum* RS5 produced comparable strength of extracellular proteolytic activity in alkaline condition in comparison to *L. plantarum* RG14, whereby there was no significant difference (*P* > 0.05) between the extracellular proteolytic activities of the three isolates in pH 8. Nevertheless, significantly higher (*P* < 0.05) extracellular proteolytic activity at pH 6.5 was detected in *L. plantarum* RS5 and *L. plantarum* RI11 among all the tested LAB isolates, with an activity of approximately 17 U/mg.

The occurrence of extracellular proteolytic under broad pH conditions implied that the studied LAB isolates produced and secreted more than one extracellular protease isozymes. Thung [31] also reported similar findings, where LAB isolated from various Malaysian fermented foods demonstrated versatile proteolytic activities that active over a broad pH conditions and up to 4 different protease isozymes were identified upon purification by using Fast Protein Liquid Chromatography. Moreover, Rodarte et al. [58] reported that numerous bacteria and filamentous fungi are capable of producing proteolytic activity in more than one pH conditions. Hence, the versatile extracellular proteolytic activity of the LAB isolates could be exploited as an effective bio-agent for extracellular production of AA.

### Amino acid production profile of LAB isolates

The 17 LAB isolates that possessed versatile proteolytic system were determined subsequently for their ability to produce AA extracellularly. Results obtained in this study showed that all the 17 studied LAB isolates have the capability to produce an array of amino acids extracellularly, where increased concentration of various AA were detected (Table 2). In contrast, the AA profile of control remained unchanged throughout 24 h of incubation (Table 2), inferring that the increment of AA content was due to the presence of LAB and their versatile extracellular proteolytic activities. In comparison, the production of glutamate and valine was detected for all the studied LAB isolates, except for *L. plantarum* RI11 that only produced glutamate. Glutamate and valine productions by LAB were also reported for fish silage treated with *Lactobacillus pentosus* and *L. plantarum* [25], cassava wastes treated with *Lactobacillus delbrueckii* and *Lactobacillus coryneformis* [59], as well as cow’s milk fermented with *L. delbrueckii* subsp. *bulgaricus*, *Lactobacillus helveticus*, *Lactococcus lactis* subsp. *lactis*, and *Streptococcus thermophilus* [24]. Moreover, Vidotti et al. [26] also reported the increased glutamate content in fermented fish silage treated with *L. plantarum* but valine production was not detected.

**Table 2:** Amino acids production profile of LAB isolates

Isolates	Amino acids																	
	Asp	Glu	Asn	Ser	Gly	Thr	Arg	Ala	Tyr	Cy2	Val	Met	Trp	Phe	Ile	Leu	Lys	Pro
*P. pentosaceus* B12m9	**–**	**++**	**–**	**+**	**+**	**+**	**–**	**+**	**+**	**+**	**+**	**+**	**–**	**–**	**+**	**+**	**–**	**+**
*P. pentosaceus* TB-1	**+**	**++**	**+**	**+**	**+**	**+**	**–**	**+**	**–**	**+**	**+**	**+**	**–**	**+**	**+**	**++**	**–**	**+**
*P. pentosaceus* TL-3	**–**	**++**	**–**	**+**	**+**	**++**	**–**	**–**	**–**	**+**	**+**	**+**	**–**	**+**	**++**	**+++**	**–**	**++**
*P. pentosaceus* TP-3	**–**	**+++**	**–**	**+**	**++**	**++**	**–**	**+**	**–**	**+**	**++**	**+**	**–**	**+**	**+**	**+++**	**–**	**+**
*P. pentosaceus* TP-4	**–**	**+**	**–**	**–**	**+**	**+**	**–**	**–**	**–**	**+**	**+**	**+**	**–**	**+**	**+**	**+**	**–**	**+**
*P. pentosaceus* TP-8	**–**	**++**	**–**	**+**	**+**	**+**	**–**	**+**	**–**	**+**	**+**	**+**	**–**	**–**	**+**	**–**	**–**	**+**
*P. acidilactici* TB-2	**–**	**++**	**–**	**+**	**+**	**++**	**–**	**+**	**–**	**+**	**+**	**+**	**–**	**+**	**+++**	**+++**	**–**	**++**
*P. acidilactici* TP-6	**+**	**+**	**–**	**–**	**+**	**+**	**–**	**–**	**–**	**+**	**+**	**–**	**+**	**+++**	**+**	**–**	**–**	**++**
*L. plantarum* TL-2	**–**	**+**	**–**	**–**	**–**	**–**	**–**	**–**	**–**	**–**	**+**	**–**	**–**	**–**	**–**	**–**	**–**	**–**
*L. plantarum* TP-2	**–**	**+**	**–**	**–**	**+**	**+**	**–**	**–**	**–**	**–**	**+**	**–**	**–**	**–**	**+**	**++**	**–**	**–**
*L. plantarum* TP-5	**–**	**+**	**–**	**–**	**+**	**–**	**–**	**–**	**–**	**–**	**+**	**–**	**–**	**–**	**–**	**–**	**–**	**+**
*L. plantarum* TL-1	**–**	**++**	**–**	**–**	**+**	**+**	**–**	**–**	**–**	**–**	**+**	**–**	**–**	**–**	**–**	**–**	**–**	**+**
*L. plantarum* I-UL4	**–**	**++**	**–**	**–**	**–**	**–**	**–**	**–**	**–**	**+**	**+**	**–**	**–**	**+**	**–**	**–**	**+**	**–**
*L. plantarum* RI11	**–**	**+**	**–**	**–**	**–**	**–**	**–**	**–**	**–**	**–**	**–**	**–**	**–**	**–**	**–**	**–**	**–**	**–**
*L. plantarum* RG11	**–**	**++**	**–**	**–**	**+**	**–**	**–**	**–**	**–**	**–**	**+**	**–**	**–**	**–**	**–**	**–**	**–**	**–**
*L. plantarum* RG14	**–**	**+**	**–**	**–**	**+**	**–**	**–**	**–**	**–**	**–**	**+**	**–**	**–**	**–**	**–**	**–**	**–**	**–**
*L. plantarum* RS5	**–**	**+**	**–**	**–**	**+**	**–**	**–**	**–**	**–**	**–**	**+**	**+**	**–**	**–**	**–**	**–**	**–**	**+**
Control	0	0	0	0	0	0	0	0	0	0	0	0	0	0	0	0	0	0

‘+’ indicates 0.1–50 mg/L increment; ‘++’ indicates 50.1–100 mg/L increment; ‘+++’ indicates > 100 mg/L increment; ‘-’ indicates 0.1–50 mg/L decrement; ‘--’ indicates 50.1–100 mg/L decrement; ‘---’ indicates > 100 mg/L decrement; ‘0’ indicates neither significant (*P* < 0.05) increment nor decrement

In contrast, the studied LAB isolates did not show the ability to produce arginine, whereby all the tested LAB isolates displayed a reducing arginine profile. Consumption of arginine by *Lactobacillus* sp. has been reported by Lee et al. [60], where a drastic reduction of arginine content was observed, suggesting that arginine plays a crucial role in ensuring the survival of LAB [61]. Manca de Nadra et al. [62] reported that some LAB were capable of degrading L-arginine via Arginine Dihydrolase (ADI) pathway to produce additional energy. Despite contradictory finding reported by Simova et al. [24], where increased arginine content was found in cow’s milk fermented with *L. delbrueckii* subsp. *bulgaricus*, *L. lactis* subsp. *lactis*, and *S. thermophilus*, yet the increment was relatively low. Surprisingly, results obtained in this study demonstrated *Lactobacillus* sp. has great requirement for serine instead of arginine, implying that serine could be one of the essential AA for the growth of *Lactobacillus* sp. Depletion of serine in *L. plantarum* could be attributed to the action of serine dehydratase that responsible for the deamination of serine into ammonia and pyruvate and ultimately into organic acids [63].

Generally, results obtained in the current study show that each LAB isolate exhibited different production profile of AA despite they belong to the same species, suggesting that the AA production was strain dependent. For instances, *P. pentosaceus* TB-1 produced comparatively vast quantities of glutamate and leucine, while *P. pentosaceus* TP-3 produced relatively high amount of glycine, threonine and valine in addition to glutamate and leucine. Nevertheless, profound differences between the AA production profiles of LAB from different genus were noted in this study (Table 2). Comparing between the two main LAB genera of LAB employed in present study, *Pediococcus* sp. demonstrated relatively higher AA production ability, whereby all the *Pediococcus* strains produced between 10 and 14 types of AA and all the *Lactobacillus* strains produced between 1 and 6 types of AA in lower quantity (Table 2).

Despite *Lactobacillus* strains used in the present study exhibited comparatively higher extracellular proteolytic activity as compared to the *Pediococci*, yet the AA production profile was in a dramatic reverse feature. This might be attributed to the production of different proteolytic enzymes by different LAB species [44]. The release of AA from proteins relies heavily on the action of aminopeptidases that responsible for the cleavage of AA from N-terminus of peptides to liberate free AA. *Pediococcus* sp. were reported to exhibit high aminopeptidase activity in a study conducted by Vafopoulou-Mastrojiannaki et al. [64], where all the 10 *Pediococci* showed relatively high aminopeptidase activity. Similarly, Carafa et al. [65] also demonstrated that four *P. pentosaceus* isolated from spontaneously fermented mountain cheese exhibited superior aminopeptidase activity from 300 to 750 U/mg, whereas the aminopeptidase activity of the *L. plantarum* was comparably lower. In addition, Mtshali et al. [66] showed that various peptidases genes including *pepC*, *pepI*, *pepN*, *pepM* and *pepT* were present in all the tested *P. pentosaceus* and *P. acidilactici*. However, not all the tested *L. plantarum* possessed the peptidase genes.

Another possible explanation for none correlation between AA production and extracellular proteolytic activity could be attributed to the mechanisms involved in the production of AA. Generally, production of AA may occur through biodegradation pathway involving extracellular proteolysis of proteins by extracellular proteolytic enzymes or intracellular biosynthetic pathway involving biosynthesis from AA precursors. Therefore, LAB isolates might produce AA via biosynthetic pathways or a combination of both biosynthetic and biodegradation pathways instead of biodegradation pathway or biosynthetic pathway alone. Numerous studies [28, 67–69] have reported the presence of genes encoded for enzymes involved in biosynthesis of various AA in LAB. The low AA production ability of *Lactobacilli* could be due to the long-term application of *Lactobacilli* in food industry, which may lead to adaptation of the species to nutrient rich environment and subsequently resulted in lost or degenerated AA biosynthetic ability [68, 70]. Klaenhammer et al. [52] reported that *Lactobacillus* sp. possessed disrupted AA synthesis pathway; hence, their AA requirement is often compensated with pronounced proteolytic activity to obtain AA from the habitat.

### Production of feed amino acids

**Fig. 3:**
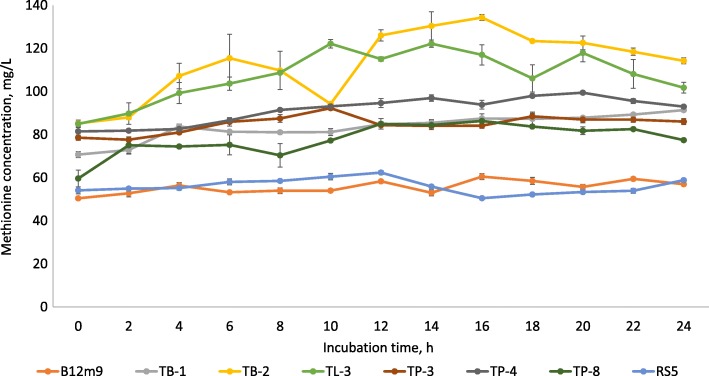
Production profile of methionine by selected LAB strains. Values are mean ± standard error mean (SEM), *n* = 3. Vertical bars represent SEM

It is noteworthy that the studied LAB isolates showed promising potential to produce various AA including feed supplement AA that are used exclusively in livestock industry such as methionine, lysine, threonine and tryptophan (Table 2). Out of the 17 tested LAB isolates, 8 of the LAB isolates including B12m9, TB-1, TL-3, TP-3, TP-4, TP-8, TB-2 and RS5 were capable of producing methionine (Table 2). The methionine production profile of the 8 LAB isolates is depicted in Fig. 3, whereby TB-1, TL-3, TP-3, TP-8 and RS5 isolates produced methionine from the beginning of cultivation until 12–14 h of incubation, whereas the methionine production by B12m9 and TB-2 isolates were extended until 16 h of incubation and 20 h of incubation for TP-4 isolate. Thereafter, the methionine content reduced slowly or remained plateau. The highest production of methionine was recorded by *P. acidilactici* TB-2, followed by *P. pentosaceus* TL-3 and *P. pentosaceus* TP-8 with 49.14 mg/L, 37.23 mg/L and 26.71 mg/L net increment of methionine content respectively. Methionine production was also reported by Simova et al. [24] in cow’s milk fermented with *L. delbrueckii* subsp. *bulgaricus*, *L. helveticus*, *L. lactis* subsp. *lactis*, and *S. thermophilus*, yet the production was relatively low with merely 4.2 mg/L of methionine production was detected. The methionine production kinetic parameter by the 8 LAB producer strains are shown in Table 3. Among the 8 methionine producing LAB, *P. pentosaceus* TL-3 demonstrated the highest methionine productivity of 3.72 mg/L/h, followed by *P. acidilactici* TB-2 (3.07 mg/L/h). Despite *P. acidilactici* TB-2 produced the highest amount of net methionine yet the productivity was slightly lower than *P. pentosaceus* TL-3 due to the longer incubation time required to achieve the highest methionine production.

**Table 3:** Kinetic parameters of methionine and lysine productions by selected LAB strains

Isolates	Methionine			Lysine		
	T, h	Net methionine concentration, mg/L	*P*_*r*_, mg/L/h	T, h	Net lysine concetration, mg/L	*P*_*r*_, mg/L/h
*P. pentosaceus* B12m9	16	10.08	0.63	-	-	-
*P. pentosaceus* TB-1	24	20.64	0.86	-	-	-
*P. pentosaceus* TL-3	10	37.21	3.72	-	-	--
*P. pentosaceus* TP-3	10	13.72	1.37	-	-	-
*P. pentosaceus* TP-4	20	17.99	0.90	-	-	-
*P. pentosaceus* TP-8	16	26.71	1.67	-	-	-
*P. acidilactici* TB-2	16	49.14	3.07	-	-	-
*L. plantarum* RS5	12	8.28	0.69	-	-	-
*L. plantarum* I-UL4	-	-	-	6	7.44	1.24

**Fig. 4:**
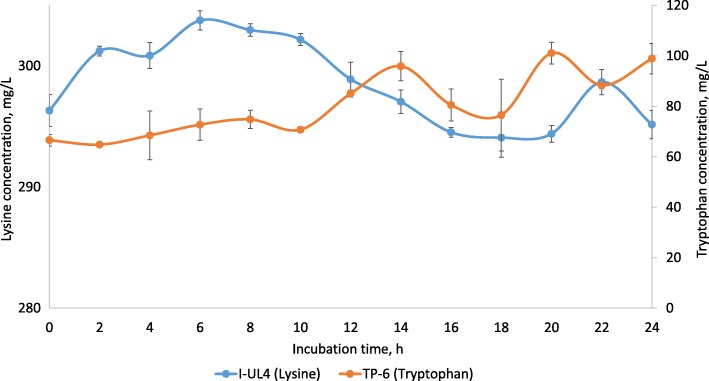
Production profiles of lysine and tryptophan by selected LAB strains. Values are mean ± standard error mean (SEM), *n* = 3. Vertical bars represent SEM

In comparison, lysine production was only detected in *L. plantarum* I-UL4 with relatively low percentage of increment (2.5%), whereby the lysine content was increased from 296.31 mg/L at the beginning of cultivation to 303.76 mg/L at 6 h of incubation. The net increment of lysine was 7.44 mg/L (Fig. 4) and the lysine productivity was 1.24 mg/L/h (Table 3). Odunfa et al. [71] also reported extracellular lysine production by *Lactobacillus* sp., whereby up to 86 mg/L of extracellularlysine production was detected. The lysine production recorded by *L. plantarum* I-UL4 in the current study is relatively low as compared to those reported by Odunfa et al. [71].

Among the LAB isolates, tryptophan production was only detected in *P. acidilactici* TP-6, whereby the tryptophan content was increased slowly from the beginning of incubation until 8 h of incubation. Thereafter, the production of tryptophan increased drastically up to 14 h, followed by a steep reduction until 18 h of incubation. Subsequently, the tryptophan content was increased tremendously to approximately 100 mg/L and maintained until the end of incubation (Fig. 4). Up to 51.84% increment of tryptophan content was detected at 20 h of incubation, which was equivalent to a net increment of 34.51 mg/L and a productivity of 1.73 mg/L/h (Table 4). Tryptophan production by *L. delbrueckii* subsp. *bulgaricus* in cow’s milk was reported by Simova et al. [24] but the production was relatively low (7.4 mg/L of tryptophan was produced). Similarly, the production of tryptophan recorded by *P. acidilactici* TP-6 in the current study was approximately 10 fold higher as compared to the tryptophan production by *Lactobacilli* in a study conducted by Tarek and Hesham [72]. Contradictorily, tryptophan production was not detected in fish silage treated with *L. plantarum* [26].

**Table 4:** Kinetic parameters of threonine and tryptophan productions by selected LAB strains

Isolates	Threonine			Tryptophan		
	T, h	Net threonine concentration, mg/L	*P*_*r*_, mg/L/h	T, h	Net tryptophan concentration, mg/L	*P*_*r*_, mg/L/h
*P. pentosaceus* B12m9	22	30.20	1.37	-	-	-
*P. pentosaceus* TB-1	24	41.31	1.72	-	-	-
*P. pentosaceus* TL-3	10	55.80	5.58	-	-	-
*P. pentosaceus* TP-3	16	52.75	3.30	-	-	-
*P. pentosaceus* TP-4	10	15.46	1.55	-	-	-
*P. pentosaceus* TP-8	16	18.58	1.16	-	-	-
*P. acidilactici* TB-2	18	58.41	3.25	-	-	-
*P. acidilactici* TP-6	18	29.79	1.66	20	34.51	1.73
*L. plantarum* TL-1	16	11.55	0.72	-	-	-
*L. plantarum* TP-2	22	8.11	0.37	-	-	-

**Fig. 5:**
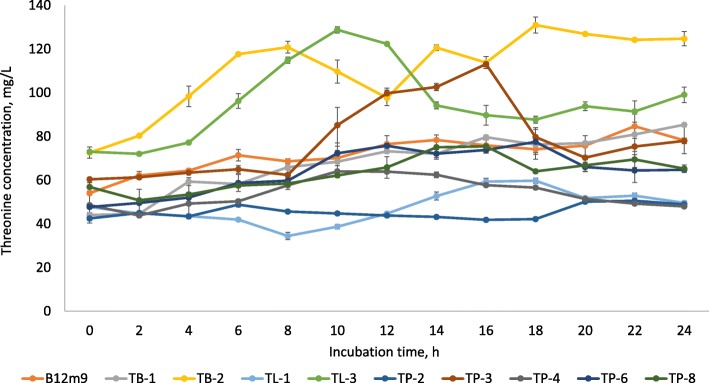
Production profile of threonine by selected LAB strains. Values are mean ± standard error mean (SEM), *n* = 3. Vertical bars represent SEM

Interestingly, 59% of the studied LAB isolates demonstrated relatively prodigious ability to produce threonine with highest production recorded by *P. acidilactici* TB-2 and *P. pentosaceus* TL-3 (Fig. 5). The former produced a net increment of 58.41 mg/L, whereby the threonine content was increased from 72.58 mg/L from beginning of incubation up to 130.99 mg/L at 18 h of incubation. Meanwhile, *P. pentosaceus* TL-3 produced 55.80 mg/L of net threonine, resulting in a final threonine concentration of 128.78 mg/L at 10 h of incubation. Contradictory, the threonine production by *L. lactis* subsp. *lactis*, *S. thermophilus* [24] and *Lactobacillus* sp. [72] was comparatively low, ranging from 0.4–8 mg/L. Among the 10 threonine producing LAB, *P. pentosaceus* TL-3 demonstrated the highest threonine productivity (*P*_*r*_) of 5.58 mg/L/h (Table 4). Despite *P. acidilactici* TB-2 produced higher amount of threonine as compared to *P. pentosaceus* TL-3, yet the time required by the isolate to achieve the highest production was much longer than *P. pentosaceus* TL-3. Consequently, the productivity of *P. acidilactici* TB-2 was nearly 1.8 fold lower than the threonine productivity of *P. pentosaceus* TL-3.

## Conclusions

All the 17 studied LAB isolates possessed versatile extracellular proteolytic activities that active over a broad pH range. The highest proteolytic activity of 15.76 U/mg and 19.42 U/mg was achieved by *L. plantarum* RG14 at pH 5 and pH 8 respectively, while *L. plantarum* RS5 showed the highest proteolytic activity at pH 6.5 (17.22 U/mg). Generally, the studied LAB isolates have the capability of producing an array of AA including a few major feed supplement AA such as methionine, lysine, threonine and tryptophan. However, the AA production was strain dependent, where different isolates exhibited different preference and efficiency in AA production. Furthermore, *Pediococcus* sp. demonstrated greater AA production ability in comparison to *Lactobacillus* sp. despite the proteolytic activity was a completely reversal manner, implying the proteolytic activity did not correlate with the AA production capability. Additionally, the production of AA by the LAB isolates might occur through biosynthetic pathway or a combination of biosynthetic and biodegradation pathways, which will require further study to elucidate the mechanism of AA production mediated by studied LAB. In comparison, *P. pentosaceus* TL-3 recorded the highest methionine and threonine productivity of 3.72 mg/L/h and 5.58 mg/L/h respectively, whereas *L. plantarum* I-UL4 demonstrated a lysine productivity of 1.24 mg/L/h and *P. acidilactici* TP-6 achieved up to 1.73 mg/L/h of tryptophan productivity. Obviously all the 17 studied LAB isolates exhibited versatile extracellular proteolytic activities and hence they possessed vast potential as a bio-agent for the productions of various bioactive peptides and AA extracellularly as feed supplements.

## Abbreviations

AA: Amino acids
ANOVA: Analysis of variance
CFS: Cell-free-supernatant
CFU/mL: Colony forming unit per millilitre
FMOC: 9-fluorenylmethyl chloroformate
GRAS: Generally Recognised as Safe
HPLC: High performance liquid chromatograph
*L. plantarum*: *Lactobacillus plantarum*
LAB: Lactic acid bacteria
*Lactobacillus* sp.: *Lactobacillus* species
MRS: De Man, Rogosa and Sharpe
OPA: O-phthalaldehyde
*P. acidilactici*: *Pediococcus acidilactici*
*P. pentosaceus*: *Pediococcus pentosaceus*
*Pediococcus* sp.: *Pediococcus* species
SMAWD: Skim milk agar well diffusion
